# Proteomics and Organoid Culture Reveal the Underlying Pathogenesis of Hashimoto’s Thyroiditis

**DOI:** 10.3389/fimmu.2021.784975

**Published:** 2021-12-02

**Authors:** Hui Xiao, Jianqing Liang, Sunqiang Liu, Qiongyue Zhang, Famin Xie, Xingyu Kong, Shanshan Guo, Ruwen Wang, Rong Fu, Zhiqi Ye, Yun Li, Shuang Zhang, Li Zhang, Keneilwe Kenny Kaudimba, Ru Wang, Xingxing Kong, Bing Zhao, Xuqin Zheng, Tiemin Liu

**Affiliations:** ^1^ Human Phenome Institute, Fudan University, Shanghai, China; ^2^ State Key Laboratory of Genetic Engineering, School of Life Sciences, Zhongshan Hospital, Fudan University, Shanghai, China; ^3^ Department of Endocrinology and Metabolism, the First Affiliated Hospital of Nanjing Medical University, Nanjing, China; ^4^ Shanghai Key Laboratory of Metabolic Remodeling and Health, Institute of Metabolism & Integrative Biology, Fudan University, Shanghai, China; ^5^ Division of Endocrinology and Metabolism, Department of Internal Medicine, Huashan Hospital, Shanghai Medical College, Fudan University, Shanghai, China; ^6^ School of Kinesiology, Key Laboratory of Exercise and Health Sciences of Ministry of Education, Shanghai University of Sport, Shanghai, China; ^7^ Shanghai Frontiers Science Research Base of Exercise and Metabolic Health, Shanghai, China; ^8^ Department of General Surgery, the First Affiliated Hospital of Nanjing Medical University, Nanjing, China; ^9^ Department of Kinesiology, Harbin Sport University, Harbin, China

**Keywords:** Hashimoto’s thyroiditis, autoimmune diseases, proteomics, organoid, pathogenesis

## Abstract

Hashimoto’s thyroiditis (HT) is an autoimmune disease, and its incidence continues to rise. Although scientists have studied this disease for many years and discovered the potential effects of various proteins in it, the specific pathogenesis is still not fully comprehended. To understand HT and translate this knowledge to clinical applications, we took the mass spectrometric analysis on thyroid tissue fine-needle puncture from HT patients and healthy people in an attempt to make a further understanding of the pathogenesis of HT. A total of 44 proteins with differential expression were identified in HT patients, and these proteins play vital roles in cell adhesion, cell metabolism, and thyroxine synthesis. Combining patient clinical trial sample information, we further compared the transient changes of gene expression regulation in HT and papillary thyroid carcinoma (PTC) samples. More importantly, we developed patient-derived HT and PTC organoids as a promising new preclinical model to verify these potential markers. Our data revealed a marked characteristic of HT organoid in upregulating chemokines that include C-C motif chemokine ligand (*CCL*) 2 and *CCL3*, which play a key role in the pathogenesis of HT. Overall, our research has enriched everyone’s understanding of the pathogenesis of HT and provides a certain reference for the treatment of the disease.

## Introduction

Hashimoto’s thyroiditis (HT) is a common type of autoimmune disease, with far more female patients than male patients aged between 45 and 60 years (1). It has been more than a hundred years since it was first reported (2). In patients with HT, the infiltration of immune cells into thyroid tissue results in the dysfunction of thyroid follicular cells and disorder of thyroxine secretion. In addition, highly expressed thyroid peroxidase antibodies (TPOAb) and thyroglobulin antibodies (TGAb) are detected in HT patients’ serum. Although the incidence of HT in the population is as high as 5% (3) and has continued to rise over the past few decades (4), scientists do not fully comprehend the pathogenesis of this disease, and it is generally recognized that a variety of genetic and environmental factors lead to the occurrence of HT (5). After genetic investigations in HT patients’ family members, it has been found that the probability of HT in monozygotic twins is much higher than that of dizygotic twins (6). Several susceptibility loci were found and identified in association with autoimmune disease or autoimmune thyroid disease by genome-wide association studies (GWAS) (7, 8). Recent studies have shown that single-nucleotide polymorphisms in multiple genes such as monocyte chemoattractant protein (*MCP*)1, interleukin (*IL*)1, and transforming growth factor beta (*TGFB*)1 are involved in genetic predisposition to autoimmune diseases, particularly HT (9–11). In addition to intrinsic factors, excessive iodine intake also results in autophagy of thyroid follicular cells and induces HT (12). Chemokines are a family of small, secreted, and structurally related cytokines with a crucial role in inflammation and immunity (13). Considering the basic role that chemokines have in orchestrating the movement of lymphocytes and the formation of lymphoid structures, it is not surprising that chemokines play an important role in HT pathogenesis. In previous studies, scientists found that many chemokines were elevated in the serum and thyroid levels of patients with HT (14), such as *CCL2* and *CCL3*.

As an important endocrine organ of the human body, the thyroid gland affects metabolic, cardiovascular, and developmental processes (15). Thyroid hormone secretion in patients with HT is generally disturbed, which also increases the incidence rate of other diseases. Growing medical statistics indicated that HT patients have an increased risk for papillary thyroid carcinoma (PTC) than healthy people (16, 17), and patients with autoimmune thyroiditis are more likely to develop mood disorders like depression and anxiety (18, 19). Therefore, researches on HT will enrich our knowledge about the functions of the human immune system, endocrine system, and cell metabolism. However, multiple previous pieces of research focused on the role of certain proteins in immune regulation because thyroid cells produce a large number of reactive oxygen species (ROS) such as hydrogen peroxide during the synthesis of thyroxine (20, 21). Whether the disturbance of cell metabolism or other biological process affects the occurrence of HT still remains to be solved. In previous studies, most of the research on the mechanism of HT was carried out in mouse models of autoimmune deficiency. Mouse models could indeed explore HT from the perspective of the whole organism. However, the mouse model of HT still has some drawbacks. First of all, the standards for modeling mice with HT have not been consistent. Some mice were given excessive iodine intake (22), while others were injected with different concentrations of thyroid immunoglobulin (23). Secondly, the mouse model was not consistent with the symptoms of HT patients, including lymphocyte infiltration, TPO antibody positivity, and hypothyroidism (14).

*In vitro* cell culture is an important research tool to simulate human development and diseases. In the past, traditional monolayer cell culture has been widely used, but due to the lack of tissue structure and complexity, it cannot reflect the true biological process. Organoid technology reproduces the cell heterogeneity, structure, and function of original tissues by establishing powerful three-dimensional models, completely changing the *in vitro* culture tools for biomedical research. Patient-derived organoids enable researchers to reconstruct human organs and diseases in petri dishes, which brings great hope for many transformational applications, such as regenerative medicine, drug discovery, and precision medicine. Recently, organoid cultures derived from patients with PTC have been established (24, 25). However, no one has successfully established the organoids of HT. Therefore, to further understand the pathogenesis of HT, we compared differences between HT patients and healthy people with proteomics analysis. We generated an HT organoid model to assess the treatment prediction potential and find new methods of prevention and treatment of HT.

## Results

### Overview of Sample Preparation and Analysis of Proteomic Profiling of Hashimoto’s Thyroiditis and Healthy Control

**Figure 1:**
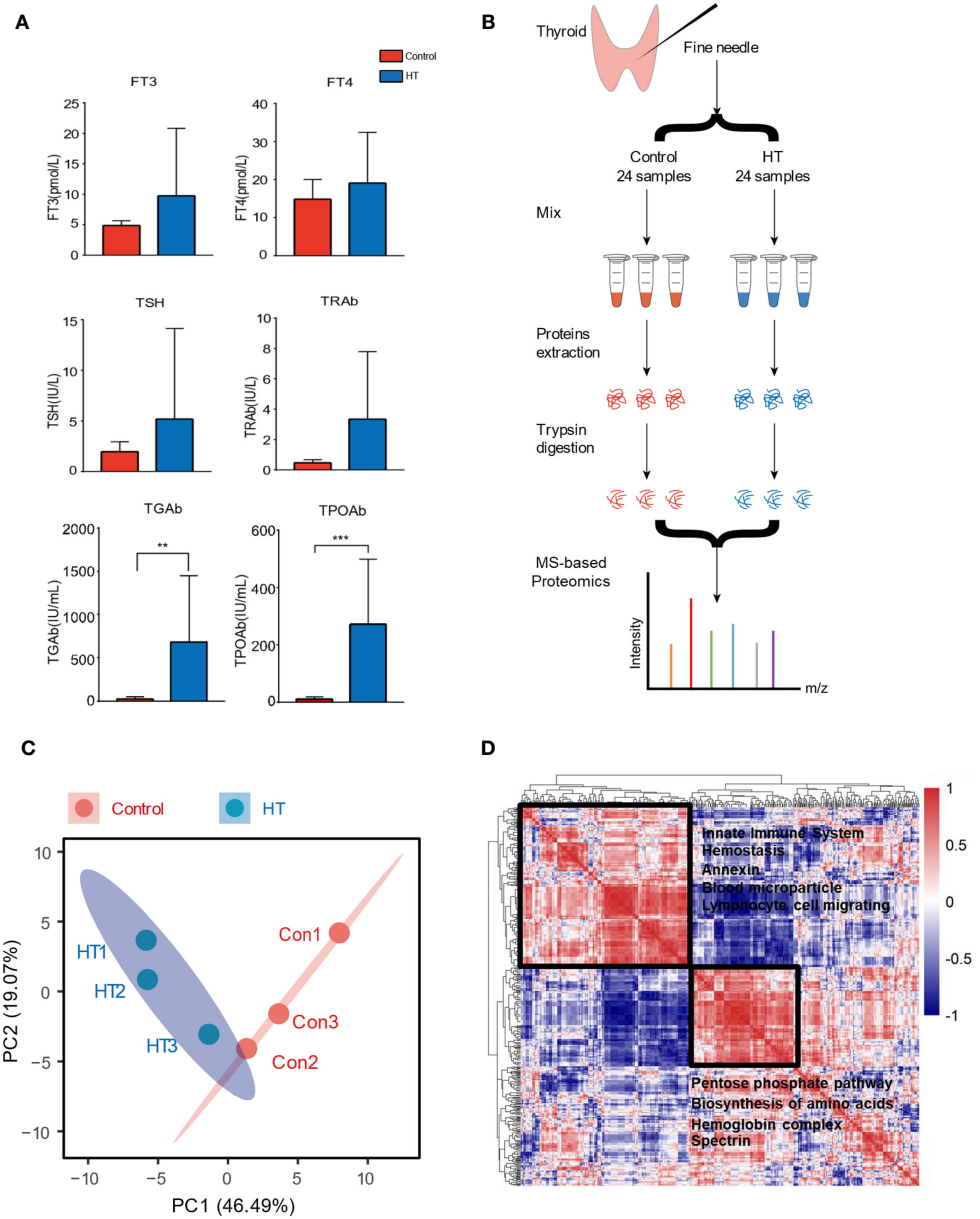
Overview of sample preparation and result analysis presentation of proteomics. **(A)** Comparison of antibody concentration in serum of Hashimoto’s thyroiditis (HT) and healthy controls. **(B)** Here, 24 patients and 24 healthy people were divided into two groups, the samples were acquired by fine-needle aspiration, and eight samples were mixed into one tube for subsequent mass spectrometry (MS). **(C)** Principal component analysis (PCA) biplot of protein concentration level; red dots represent the cluster Control, while blue dots represent the cluster HT. The first two axes accounted for 65% of variance. **(D)** Global correlation map of proteins generated by clustering the Pearson correlation coefficients of all possible protein combinations. The abundance of proteins with common regulation correlates across samples, and they therefore form a cluster. Prominent clusters are annotated with functional terms obtained from bioinformatics enrichment analysis. The inset shows the color code for Pearson correlation coefficients. Error bars show the mean ± SEM. Asterisks signify significant differences using one-way ANOVA, **P < 0.01; ***P < 0.001.

In the endocrinology department, patients whose serum was found with positive TPO antibody (TPOAb >34.0 IU/ml or TGAb >115.0 IU/ml) were diagnosed with HT, while those with pure thyroid nodules were considered healthy control. There is no significant difference in body mass index (BMI) of these patients compared with healthy people, but the concentrations of antibodies such as TGAb and TPOAb in HT patients increased (**Figure 1A** and **Table 1**). To explore the difference in protein expression of thyroid in patients with HT, we used fine-needle aspiration biopsy to extract tissue fluid from the thyroid site in 24 HT patients and 24 healthy people who had pure thyroid nodules.

**Table 1:** Clinical characteristics of HT samples.

	Control	HT	*P*-value
	*N = 24*	*N = 24*	
F	19	23	
M	5	1	
Age (years)	48.54 ± 11.67	41.38 ± 12.98	0.050
BMI (kg/m^2^)	22.42 ± 3.40	23.03 ± 2.31	0.554
FT3 (pmol/l)	4.86 ± 0.81	9.76 ± 11.07*	0.041
FT4 (pmol/l)	14.89 ± 5.11	25.83 ± 26.16	0.055
TSH (MIU/l)	1.96 ± 0.98	4.32 ± 8.38	0.183
TPOAb (IU/ml)	10.26 ± 7.06	408.10 ± 238.75***	<0.001
TGAb (IU/ml)	21.43 ± 29.12	982.32 ± 1194.77**	0.001
TRAb (IU/L)	1.82 ± 4.38	3.07 ± 4.32	0.464

HT, Hashimoto’s thyroiditis; F, female; M, male; BMI, body mass index; FT3, free triiodothyronine; FT4, free thyroxine; TSH, thyroid-stimulating hormone; TRAb, thyrotropin receptor antibody; TGAb, anti-thyroglobulin antibody; TPOAb, thyroid peroxidase antibody.

Plus–minus values are means ± SD.

*P < 0.05, **P < 0.01, ***P < 0.001.

We obtained thyroid fluid samples after obtaining patients’ consent. Due to the limitation of protein concentration and total amount in subsequent mass spectrometry (MS) experiments, the tissue fluids of eight individuals were mixed as one sample, and the experiment group (HT) and the control group (Con) were performed in triplicate (**Figure 1B**). Here, 910 proteins were obtained after analysis by MS. We analyzed the relative abundance of all proteins and presented them with principal component analysis (PCA) (**Figure 1C**). The two groups were distinguished to form two sections, which indicates that there is a significant difference in protein expression in thyroid tissue sites between HT patients and healthy people with thyroid nodules. Next, we investigated the relationship between different proteins to functionally explain the co-regulatory clusters between proteins or clinical parameters. The global protein correlation map highlights two main clusters of proteins. For instance, the immune regulation terms such as innate immune system and lymphocyte cell migrating were selectively enriched in the largest cluster. And the other cluster was enriched in proteins relating to the pentose phosphate pathway, biosynthesis of amino acids, and hemoglobin complex (**Figure 1D**).

### Significant Differences in Protein Expression Between Hashimoto’s Thyroiditis Patients and Healthy People

**Figure 2:**
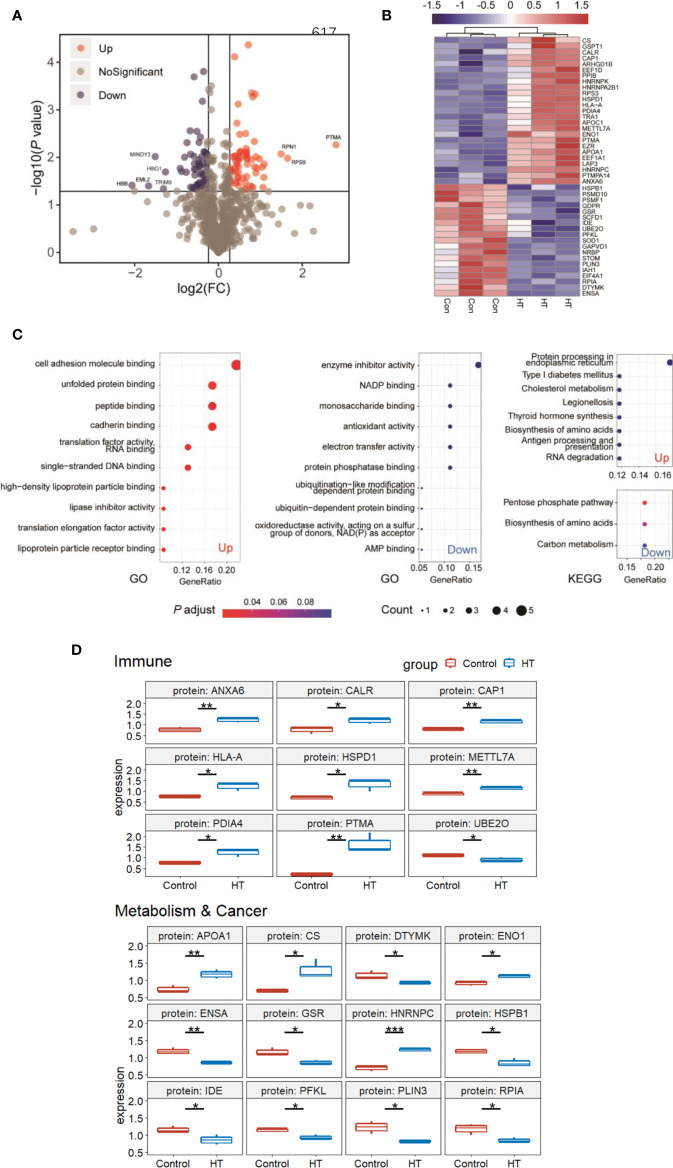
Significant differences in protein expression between Hashimoto’s thyroiditis (HT) patients and healthy people. **(A)** Volcano plot of proteomic data. Volcano plots are depicted with the fold change of each protein, and the *P*-value was calculated by performing t-test. Red circles show proteins that have significant increases. Blue circles show proteins that have significant decreases. Gray circles are proteins without any differences. **(B)** Heat map of the 44 differentially expressed protein (DEPs) information. **(C)** GO and KEGG pathway analysis of 26 upregulated and 18 downregulated genes. **(D)** Summary of DEPs in key functional groups that show differences between HT and Control. DEPs related to the immune system and metabolism&cancer are shown in boxplots. Median value was marked. *P < 0.05; **P < 0.01; ***P < 0.001.

To determine the effect of differentially expressed proteins on patients, we further classified the information of the 910 proteins, screened the differentially expressed proteins between the experimental group and the control group, and set the fold change to 1.2 and *P* value less than 0.05 to draw the volcano plot; 125 proteins show a difference in the HT group *vs.* the control group (**Figure 2A**). We set the unique peptides not less than 2 for further screening and eliminated some contaminated protein peptides during the extraction process such as hemoglobin and cytoskeleton-related proteins like actin and tubulin. Ultimately, 44 proteins worthy of attention remained, of which 26 proteins were highly expressed in HT and 18 proteins were relatively low. We displayed the expression information of these differential proteins in a cluster heat map (**Figure 2B**) and performed Gene Ontology (GO) enrichment analysis and Kyoto Encyclopedia of Genes and Genomes (KEGG) enrichment analysis on the upregulated and downregulated protein (**Figure 2C**). The results indicated that upregulated proteins are related to cell adhesion, gene expression, and lipid transport by GO enrichment. The pathways of protein expression, cholesterol metabolism, thyroid hormone synthesis, and antigen presentation were enriched through KEGG pathway analysis. The GO analysis in downregulated proteins revealed that enzyme inhibitor activity, redox reactions, and ubiquitination-related protein degradation pathways were significantly enriched in HT patients. By KEGG analysis, these proteins were enriched in cellular metabolism, including the five-carbon phosphate pathway, amino acid biosynthesis, and carbon metabolism pathway. In general, upregulated proteins are related to cell adhesion, protein synthesis, thyroxine synthesis, and cellular immunity, while downregulated proteins are related to cell metabolism and protein degradation.

**Figure 3:**
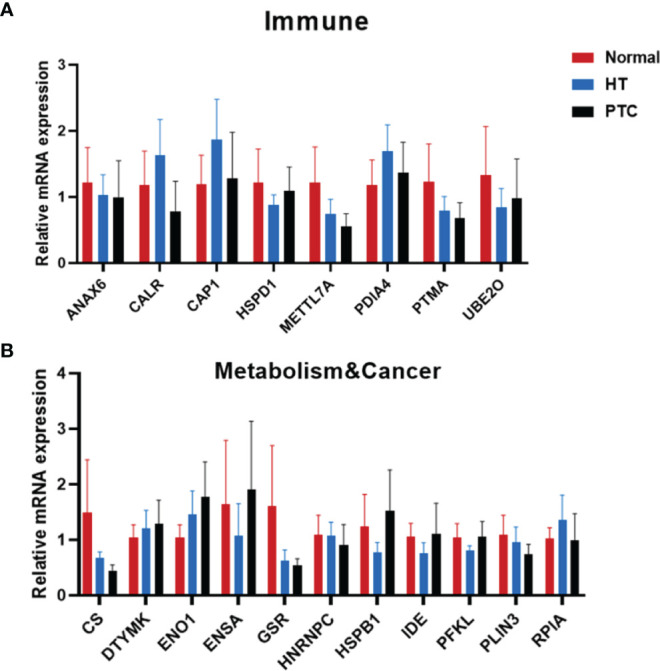
Transcriptional changes with functional groups relevant to the immune system and metabolism&cancer in thyroid tissue. Molecular changes in transcription levels between Hashimoto’s thyroiditis (HT) and Control relevant to the immune system **(A)** and metabolism&cancer **(B)** were evaluated by semiquantitative PCR [Normal, n  =  3; HT, n  =  5; papillary thyroid carcinoma (PTC), n = 6]. Error bars show the mean ± SEM.

Based on these results, we then focused on functional proteins from which we selected 44 proteins. Ultimately, we selected 21 proteins relevant to the immune system and metabolism and cancer as summarized in **Figure 2D**. A large proportion of proteins upregulated in the HT group was related to immune response, such as annexin A6 (ANXA6), calreticulin (CALR), cyclase associated actin cytoskeleton regulatory protein 1 (CAP1), major histocompatibility complex, class I, A (HLA-A), heat shock protein family D (Hsp60) member 1 (HSPD1), methyltransferase like 7A (METTL7A), prothymosin alpha (PTMA), ubiquitin conjugating enzyme E2 O (UBE2O), suggesting that the autoimmune system of patients with HT may have been disordered. However, it is not clear whether the changes in expression levels of these proteins occur at the transcriptional level or the translational level. In addition, previous research showed the relationship between HT and PTC (17, 26, 27). So, we next collected 10 cancerous and para-cancerous samples from patients with papillary thyroid cancer (**Table 2**). Seven of these patients had HT disease, and their para-cancerous samples were HT samples. The other three patients had no complications, whose para-cancerous samples were normal samples. We examined the 21 genes’ transcriptional level changes using RT-qPCR analyses in three tissues, but due to the large variation of HLA-A and APOA1 expression among individuals in one group, only 19 genes were displayed (**Figure 3**). There were no significant differences between HT and control genes, which indicates that these genes were regulated at the translational level. We further tested the expression levels of these genes in PTC (**Figure 3**). It was found that at the transcriptional level, the expression of these genes did not change between HT and PTC.

**Table 2:** Clinical characteristics of thyroid tumor samples.

Sample	Sex	Age (years)	TI-RADS	TPOAb (IU/ml)	TGAb (IU/ml)	Combined disease
PTC1	F	30	4B	517.4	504.7	HT
PTC2	F	54	4A	68.6	200.9	HT
PTC3	F	42	4B	277.8	120.0	HT
PTC4	F	31	4A	190.5	295.6	HT
PTC5	F	53	5	8.5	16.1	Non
PTC6	M	37	5	5.0	61.6	Non
PTC7	F	28	5	8.2	12.5	Non
PTC8	M	32	4A	104.4	NA	HT
PTC9	F	34	4C	239.7	>4,000	HT
PTC10	F	25	5	92.7	38.1	HT

F, female; M, male; PTC, papillary thyroid carcinoma; TI-RADS, Thyroid Imaging Reporting and Data System; TGAb, anti-thyroglobulin antibody; TPOAb, thyroid peroxidase antibody; NA, not available.

### *In Vitro* Self-Renewal and Organoid Formation Derived From Patients With Hashimoto’s Thyroiditis and Papillary Thyroid Carcinoma

**Figure 4:**
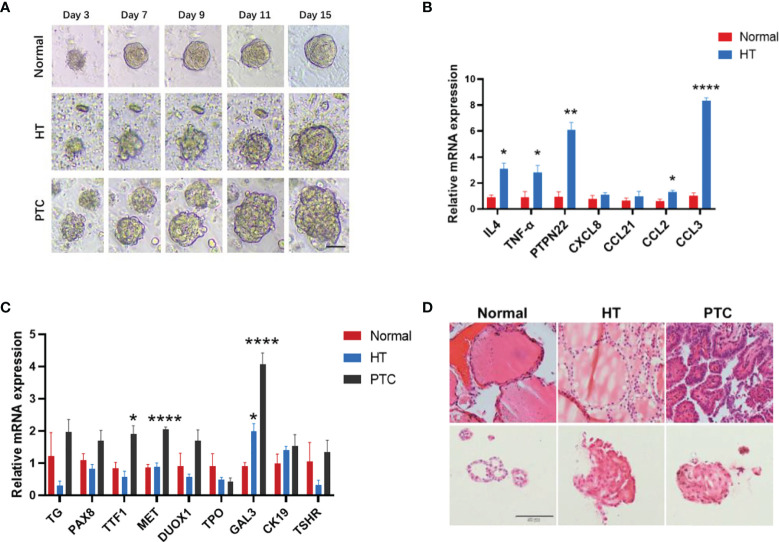
Organoid cultures derived from patients with Hashimoto’s thyroiditis (HT) and papillary thyroid carcinoma (PTC). **(A)** Time-lapse imaging sequence of Control, HT, and PTC organoids. Scale bar, 100 μm. **(B)** Quantitative PCR (qPCR) analysis of the HT and chemokine characterization marker mRNA level in thyroid organoids (Normal, n = 4; HT, n = 4). **(C)** qPCR analysis of the HT characterization marker mRNA level in thyroid organoids (Normal, n = 3; HT, n = 5; PTC, n = 6). All data are presented as mean ± SEM. **(D)** H&E staining of the organoids and their corresponding tissues, respectively. Scale bar, 50 μm. Error bars show the mean ± SEM. *P < 0.05; **P < 0.01; ****P < 0.00001.

Due to the difficulty in obtaining clinical samples and constructing mouse models, we established organoids for HT and PTC to facilitate the study of these two diseases. Patients’ thyroid tissues were dissociated into single cells and cell clumps using mechanical and enzymatic digestion. Then, single cells were cultured in a thyroid organoid medium that supported the formation and progressive growth of thyroid organoids (**Figure 4A**). To further characterize the HT and PTC organoids, gene expression of specific thyroid markers, HT markers, and PTC markers was assessed in organoids (**Figures 4B, C**). The immune-related gene expression of *IL4*, tumor necrosis factor (*TNF*)-α, and protein tyrosine phosphatase non-receptor type 22 (*PTPN22*) was significantly higher in HT organoids compared with normal, which is consistent with the results of previous studies (28–30). Interestingly, we examined the gene expression levels of four chemokines associated with HT, and the expression levels of these chemokines all showed an upward trend in HT, among which the chemokines *CCL2* and *CCL3* were significantly upregulated. This suggested that the HT organoid from the HT patient well represents the characteristics of the HT tissue. Histological examination using hematoxylin and eosin (H&E) staining also showed that the HT organoids displayed HT-like morphology with nuclear and cellular atypia, a similar morphology to that of the components in their original tissues (**Figure 4D**). More interestingly, the expression of GAL3, a biomarker of PTC disease (31), gradually increased in HT and PTC and significantly increased in PTC. High positive expression of GAL3 in HT may have a role in the cellular transformation to a cancerous cell with PTC feature when overexpression is continuous (32). These results suggests that HT may have similar characteristics with PTC and may progress to PTC.

**Figure 5:**
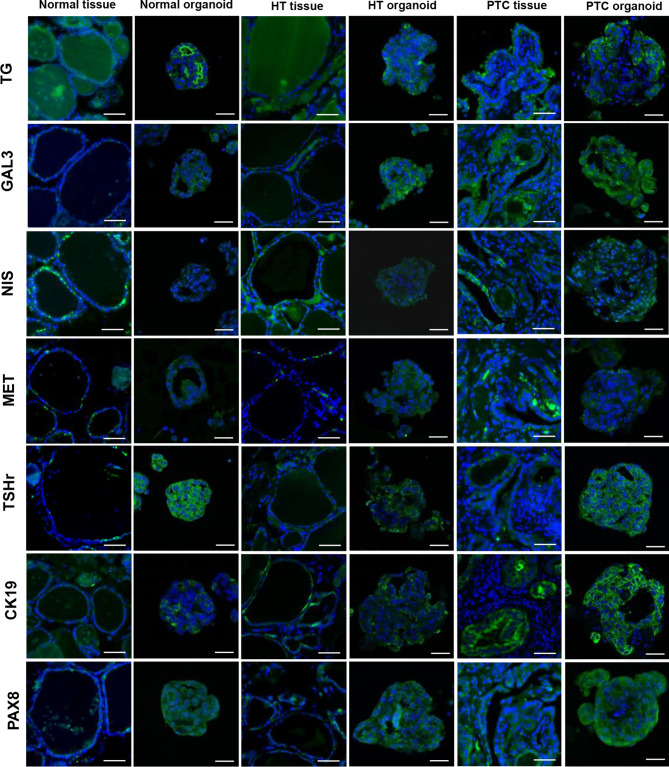
Representative images of thyroids organoids subjected to immunofluorescence analysis for thyroid-specific markers, papillary thyroid carcinoma (PTC) markers. Organoids were cultured for 15 days before fixation and staining with Alexa-488 phalloidin and 4′,6-diamidino-2-phenylindole (DAPI). Scale bars, tissues = 50 µm, organoid = 20 µm. Markers indicated on the left side are shown as a green fluorescent signal. Nuclei are shown as a blue fluorescent signal.

In addition, to further characterize the HT organoids, we performed immunofluorescence (IF) analyses of marker expression in thyroid organoids and tissues. The results showed that the marker expression profiles were consistent between thyroid tissues and their derived organoids (**Figure 5**). Combining gene expression data and IF, we showed that our HT organoids are very similar to HT tissue, thus making sense of its use as an *in vitro* model of basic and translational research in HT.

### Further Confirmation of Hashimoto’s Thyroiditis-Associated Gene Alterations in Organoids

**Figure 6:**
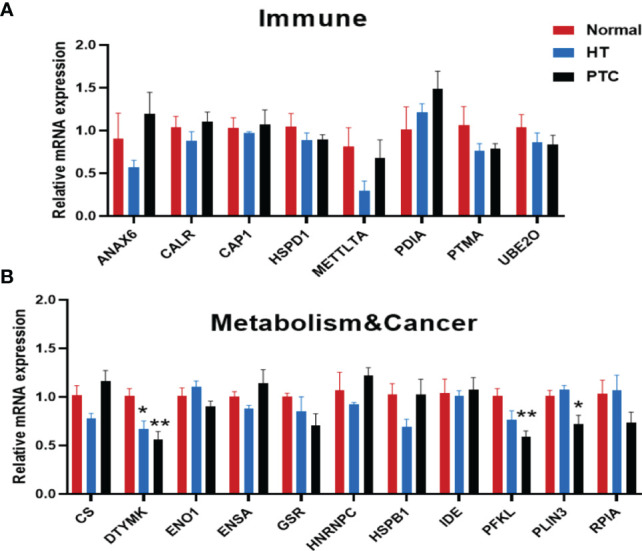
RT-qPCR results of genes relevant to the immune system **(A)** and metabolism & cancer **(B)** in thyroid organoids from Normal, HT and PTC. Thyroid organoids were collected 15 days after plating and RNA prepared for RT-qPCR analysis. (Normal, n = 5; HT, n = 3; PTC, n = 6). Error bars show the mean ± SEM. *P < 0.05; **P < 0.01.

Patient-derived organoids have the potential to be used as preclinical models for confirming the proteome results. We performed RT-qPCR assays to examine gene expression levels in thyroid organoids that showed marked differences between the HT and control groups summarized by proteome (**Figures 6A, B**). Interestingly, similar to *CK19*, the expression of deoxythymidylate kinase (DTYMK) in HT and PTC organoids also gradually decreased compared with normal organoids (**Figure 6B**). DTYMK is a nuclear DTYMK, involved in the pathway deoxythymidine triphosphate biosynthesis, which is part of pyrimidine metabolism. In hepatocellular carcinoma, DTYMK expression predicts prognosis and chemotherapeutic response and correlates with the immune infiltration (33). In the PTC organoid, citrate synthase (CS) expression was increased, while phosphofructokinase, liver type (PFKL) and perilipin 3 (PLIN3) expression decreased. PLIN3, which belongs to the perilipin family, is considered to be involved in lipid droplet formation and the storage of lipids in cells (34). PLIN3 serves as a potential diagnostic and prognostic biomarker in renal cell carcinoma, and its expression is upregulated in renal cell carcinoma cells and tissues (35).

## Discussion

In this study, we compared the thyroid proteome map between HT and healthy people, and 912 proteins can be quantified and over 120 proteins alter in HT disease condition. Through the bioinformatics analysis, we found that these immune-related genes (ANXA6, CALR, CAP1, HLA-A, HSPD1, METTL7A, PTMA, and UBE2O) were increasing in HT thyroid, which may be candidates of HT pathogenic gene. Among them, the protein level of PTMA is the one that has positive HT at its highest form. Previous research has shown that PTMA was initially isolated from fresh rat thymus (36). Accordingly, inside the cell, PTMA is implicated in crucial intracellular circuits and may serve as a surrogate tumor biomarker, but when found outside the cell, it could be used as a therapeutic agent for treating immune system deficiencies (37). In addition, many studies have reported that HLA-A was a susceptibility gene for autoimmune thyroid diseases (38). HSPD1, which increased in the blood of HT patients compared to controls, could very well mediate thyroid cell damage and destruction, perpetuating inflammation (39). We performed a functional enrichment analysis on proteins identified as downregulated in HT and found that these proteins were mainly related to cell metabolism and protein degradation, including ubiquitination-related protein degradation, amino acid biosynthesis, and carbon metabolism pathway. Thyroid hormone was a key determinant of cell metabolism, regulating the pathways of carbohydrate, lipid, and protein metabolism (40). Hypothyroidism induced a hypometabolic state characterized by reduced energy expenditure, increased cholesterol levels, reduced lipolysis and gluconeogenesis, and weight gain (41). HT was a major cause of hypothyroidism, and many patients with HT eventually developed hypothyroidism (42), which may explain why cell metabolism and protein degradation process were downregulated in HT.

Furthermore, we established a culture system for HT and PTC thyroid organoids in which the thyroid cells maintain similar characteristics with thyroid tissue and a high proliferative capacity. Using thyroid organoids as a tool, we found that the expression of DTWMK gradually decreased in HT organoids and PTC organoids, indicating that DTYMK may be related to the progression of HT to PTC and may be used as a potential therapeutic target for HT. Chemokines fall in a family of small, secreted, and structurally related cytokines with a crucial role in inflammation and immunity (13), which played an important role in HT pathogenesis. In this study, we found that the chemokines *CCL2* and *CCL3* are significantly highly expressed in HT organoids, especially *CCL3* that is upregulated about eight times in HT organoids. These results suggest that the HT organoids we cultivated are consistent with the pathogenesis of the thyroid tissues and that inflammation is caused by lymphocyte infiltration.

Due to the mass spectrometry’s requirement for the total protein content of the sample, we mixed the tissue fluids of eight individuals to experiment. Because there is a certain degree of differences between each individual whose protein expression information cannot be obtained, samples from a small number of male individuals were studied with samples from women, and the effects of these individual differences could not be neglected in our experiments. On the other hand, the process of mixing samples from different individuals also has an advantage in the experiment in that it can effectively avoid data deviations caused by a single individual to the entire group. In addition, in the process of screening protein collections, we adopted a method with the number of unique peptides greater than or equal to 2 and neglected some proteins with only one unique peptide being detected. These proteins may also be important markers like small RNA binding exonuclease protection factor La (*SSB*), it is related to systemic lupus erythematosus, which is another common autoimmune disease. This shows that these autoimmune diseases may have some of the same expression profiles.

In summary, we profiled thyroid aspiration biopsy proteome maps in HT patients and successfully became the first to establish a culture system for HT thyroid organoids in which the thyroid cells maintain a high proliferative capacity. Additional research is needed to further explore and confirm the clinical application of this procedure before providing any basis for clinical decisions.

## Materials and Methods

### Human Specimens

HT fine-needle puncture and PTC tissues were obtained from The First Affiliated Hospital of Nanjing Medical University, with the approval of the Research Ethics Committee (approval no. 2017-SR-346). According to the expressed TPOAb and TGAb detected in HT patients’ serum, the patients with TPOAb >34.0 IU/ml or TGAb >115.0 IU/ml were diagnosed with HT (**Table 1**). For PTC patients, the sex, age, tumor size, and Thyroid Imaging Reporting and Data System (TI-RADS) stage were recorded when available (**Table 2**). The diagnosis of each PTC case was confirmed on routine H&E-stained slides by two pathologists. All specimens’ identities are renamed with codes instead of the patient’s name; written informed consent was provided by all patients.

### Protein Extraction

Samples were lysed in a buffer that consisted of 4% sodium dodecyl sulfate (SDS; sodium lauryl sulfonate), 100 mM Tris/HCl pH 7.6, and 0.1 M dithiothreitol (DTT), and the protein concentration was determined by a bicinchoninic acid (BCA) protein assay. An appropriate amount of protein from each sample was collected and lysed with the filter-aided sample preparation (FASP) method (43). The peptides were desalted with C18 Cartridge, lyophilized, redissolved with 40 μl dissolution buffer, and quantified by spectrophotometry method (OD280).

### Liquid Chromatography–Tandem Mass Spectrometry Analysis

Here, 100 μg of peptides were taken from each sample and labeled according to the instructions of the AB SCIEX iTRAQ Labeling Kit. Each set of labeled peptides was mixed and graded using AKTA Purifier 100. Buffer solution A: 10 mM KH_2_PO_4_, 25% ACN, pH 3.0; Buffer solution B: 10 mM KH_2_PO_4_, 500 mM KCl, 25% ACN, pH 3.0. The column was equilibrated with Buffer solution A, and each sample was loaded from the injector to the column for separation. The flow rate was 1 ml/min. The liquid phase gradient is as follows: 0–25 min, 0%–10% linear gradient Buffer B; 25–32 min, 10%–20% linear gradient Buffer B; 32–42 min, 20%–45% linear gradient Buffer B; 42–47 min, 45%–100% linear gradient Buffer B; 47–60 min, 100% Buffer B; after 60 min, 0% Buffer B. During the elution process, the absorbance at 214 nm was monitored, and the eluted fractions were collected every 1 min. After lyophilization, they were desalted with C18 Cartridge. Each fractionated sample was separated with high-performance liquid chromatography (HPLC) liquid system Easy nLC at a nanoliter flow rate. Buffer solution A: 0.1% formic acid. Buffer solution B: 84% acetonitrile with 0.1% formic acid. The column was equilibrated with 95% Buffer solution A. The sample was loaded by autosampler to the loading column (Thermo Scientific Acclaim PepMap100, 100 μm * 2 cm, nanoViper C18) and separated through the analytical column (Thermo Scientific EASY column, 10 cm, ID 75 μm, 3 μm, C18-A2) with a flow rate of 300 nl/min. The samples were chromatographed for mass spectrometry (MS) using a Q-Exactive mass spectrometer. The detection method was positive ion, the scanning range of the precursor ion was 300–1,800 m/z, the resolution of the primary mass spectrum was 70,000 at 200 m/z, the automatic gain control (AGC) target was 1e6, the maximum IT was 50 ms, and the dynamic exclusion time was 60.0 s. The mass-to-charge ratios of peptides and peptide fragments were collected according to the following method: 10 fragment spectra were acquired after a full scan (MS2 scan) with HCD MS2 Activation Type, isolation window was at 2 m/z, secondary MS resolution was 17,500 at 200 m/z, normalized collision energy was 30 eV, and underfill was 0.1%.

### Protein Identification and Quantification

The raw data for MS analysis were RAW files, before the software, and Mascot2.2 and Proteome Discoverer1.4 were used for library identification and quantitative analysis.

### Gene Ontology and Pathway Analysis

Differentially expressed proteins were screened according to the criteria that the expression fold change was more than 1.2 times (upregulation more than 1.2-fold or downregulation less than 0.83-fold) and *P*-value <0.05. The list of proteins across the two sample groups and the differentially expressed sets of proteins from all comparisons were annotated and summarized at various GO categories using the topGO and cluster profile R package.

### Organoid Culture

Fresh thyroid specimens were washed by phosphate buffered saline (PBS) and split into several smaller pieces. One piece was frozen and stored at –80°C for RNA isolation, one piece was fixed in paraformaldehyde for histopathological analysis and immunostaining, and the remaining tissues were dissociated and processed for organoid derivation. Human thyroid tissue was cut into smaller pieces (1 to –2 mm) with a surgical blade and digested with Collagenase Type I and Collagenase Type II (final concentration 100 U/ml, Thermo Fisher 17018029, 17101015) in Advanced DMEM/F-12 (Dulbecco's Modified Eagle Medium/Ham's F-12) (Thermo Fisher 12634028) with Rho kinase (ROCK) inhibitor (Y-27632, 10 µM) for 30 min at 37°C. Digested cells were collected by centrifugation at 300g for 3 min, after which the cell suspension was filtered through a 100-µm strainer. Cells were collected by centrifugation and resuspended in ∼30 µl ice-cold Matrigel (Corning, No. 356231) and plated into a 12-well plate at 37°C for 15 min. When the Matrigel was solidified, 500 µl human thyroid organoid media were added. The medium includes Advanced DMEM/F-12 (Gibco, 12634-010), fibroblast growth factor (FGF) 10 (100 ng/ml, OrganRegen, Lot 031003), B27 (2%, Thermo Fisher, 17504001), A83-01 (5 µM, PeproTech, No.9094360), N-acetylcysteine (1.25 mM, PeproTech, No. 6169116), Noggin (100 ng/ml, OrganRegen, Lot 040606), epidermal growth factor (EGF; 50 ng/ml, Peprotech, No. AF-100-15), R-spondin-1 (200 ng/ml, OrganRegen, Lot 040407), ROCK inhibitor (Y-27632 10 µM, Abcam, ab143784), and thyroid-stimulating hormone (TSH; 16 mIU/ml, Sigma-Aldrich). Organoid culture medium was added and replaced every 3 days. Organoids were passaged at a 1:2 to 1:4 dilution every 2 weeks and either dissociation using TrypLE (Thermo Fisher 12605036). ROCK inhibitor (Y-27632, 10 µM) was added to the media after passaging to prevent cell death. Organoids were frozen in freezing media (NCM Biotech, C40100) and could be recovered efficiently.

### Cell RNA Extraction and qRT-PCR

Total RNA from organoids and patient material was prepared (RNeasy Mini Kit, TIANGEN BIOTECH, DP420) following the manufacturer’s instructions. In total, 500 ng total RNA was reverse transcribed by using a Reverse transcription kit (Thermo Fisher, 4366596) with a total of 20 µl for each reaction. A quantitative polymerase chain reaction (qPCR; Accurate Biology, AG11718) was performed using SYBR^®^ Green Premix Pro Taq HS qPCR Kit according to the manufacturer’s instructions. A total of 200 ng cDNA was mixed with PCR buffer, SyberGreen, and both forward and reverse primers for genes of interest, with a total volume of 10 µl for each sample. A three-step PCR reaction was applied subsequently (QuantStudio 7, Thermo Fisher). Oligo sequences of primers used were described in the **Supplementary Table**.

### Histology and Immunofluorescence

Tissues were fixed in 4% paraformaldehyde for 24 h, embedded in paraffin, and serial sectioned at 5 μm in thickness. In the case of the organoids, Matrigel was dissolved and organoids were washed with PBS centrifuged at 200 rcf for 2 min. The resulting pellet was fixed in 4% paraformaldehyde (2 h, 4°C). Next, the organoids were embedded in 2% agarose gel (Invitrogen, 75510019), and the gel was subjected to dehydration, followed by embedding in paraffin and sectioned at 5 μm in thickness. Sections were deparaffinized in xylene for 10 min. This was followed by washing, blocking in 5% goat serum albumin blocking buffer for 20 min at room temperature, and incubation with primary antibodies at 4°C overnight. Slides were then incubated with secondary antibodies (1:500, Jackson Immune Research, 111-545-003) for 1 h, stained with 4′,6-diamidino-2-phenylindole (DAPI, Beyotime, C1002) for 10 min at room temperature, mounted using an aqueous mounting medium, and imaged using a DM6B microscope (Leica) and a FV1200 confocal microscope (Olympus). For immunofluorescence analysis, the antibodies to MET (1:50, Abclonal, A17366), GAL3 (1:50, Abclonal, A1464), TG (1:50, Proteintech, 60272-1-Ig), *CK19* (1:50, Abclonal, A0247), TSHr (1:50, Abclonal, A6781), NIS (1:50, Proteintech, 24324-1-AP), and PAX8 (1:50, Abclonal, A1009) were used to detect proteins.

### Statistical Analysis

Statistical analyses were performed by Prism GraphPad 9.0 (GraphPad Software). Unpaired t-test was used to evaluate differences between two groups, and analysis of variance (ANOVA) was used to evaluate differences among three groups. Significance was set at *P*-value <0.05.

## Data Availability Statement

The mass spectrometry proteomics data have been deposited to the ProteomeXchange Consortium (http://proteomecentral.proteomexchange.org) via the iProX partner repository (44) with the dataset identifier PXD028448.

## Ethics Statement

The studies involving human participants were reviewed and approved by The First Affiliated Hospital of Nanjing Medical University. The patients/participants provided their written informed consent to participate in this study.

## Author Contributions

TL designed study, interpreted study results, and participated in drafting and editing of manuscript. HX performed the experiments, visualization and statistical analysis, wrote and edited the manuscript. JL performed organoid experiments. SL and QZ participated in experiments and clinical data analysis. XZ, and BZ assisted in study design, participated in interpretation of results, and edited manuscript. FX, SG, RWW, ZY, YL, SZ and LZ participated in the experiments and visualization. XYK and KKK participated in the data analysis and article modification. RF, SL, and XZ assisted clinical sample collection. RW and XXK supervised the experiments, data analysis and interpretation. All authors contributed to the article and approved the submitted version.

## Funding

This research was funded by the National Key R&D Program of China (2019YFA0801900, 2018YFA0800300, 2020YFA0803800), the National Natural Science Foundation of China (31971074), Innovation Team and Talents Cultivation Program of National Administration of Traditional Chinese Medicine (ZYYCXTD-D-202001), the Shanghai Municipal Science and Technology Major Project (2017SHZDZX01), Shanghai Frontiers Science Research Base of Exercise and Metabolic Health, the National Natural Science Foundation of China (31971097), the Construction Project of High-Level Local Universities in Shanghai, China, Shanghai Municipal Science and Technology Committee of Shanghai outstanding academic leaders plan (21XD1403200), the 2020 fundamental research Fund of heilongjiang province (2020KYYWF-FC1), China Postdoctoral Science Foundation (2021M690680).

## Conflict of Interest

The authors declare that the research was conducted in the absence of any commercial or financial relationships that could be construed as a potential conflict of interest.

## Publisher’s Note

All claims expressed in this article are solely those of the authors and do not necessarily represent those of their affiliated organizations, or those of the publisher, the editors and the reviewers. Any product that may be evaluated in this article, or claim that may be made by its manufacturer, is not guaranteed or endorsed by the publisher.
