# Supplementary material for: Proteomics and Organoid Culture Reveal the Underlying Pathogenesis of Hashimoto’s Thyroiditis
Source: Front Immunol. 2021 Dec 2;12:784975. doi: 10.3389/fimmu.2021.784975 (PMC8674930; doi:10.3389/fimmu.2021.784975)
Supplement: Supplementary file 1 [file DataSheet_1.pdf]

Supplementary table. Primer list.

Primer	Sequence
H_PTMA_F	TCAGACGCAGCCGTAGACA
H_PTMA_R	GCATTCCCGTTAGCAGGGG
H_HSPD1_F	CTACTGTACTGGCACGCTCTA
H_HSPD1_R	CAACAGCTAACATCACACCTCTC
H_CS_F	TGCTTCCTCCACGAATTTGAAA
H_CS_R	CCACCATACATCATGTCCACAG
H_HNRNPC_F	TGGGCTGCTCTGTTCATAAGGG
H_HNRNPC_R	CTCGGTTCACTTTTGGCTCTGC
H_PDIA4_F	GGCAGGCTGTAGACTACGAG
H_PDIA4_R	TTGGTCAACACAAGCGTGACT
H_ANXA6_F	AGAGCTACAAGTCCCTCTACG
H_ANXA6_R	CCCACAATCAACCGTTCAAAC
H_APOA1_F	CCCTGGGATCGAGTGAAGGA
H_APOA1_R	CTGGGACACATAGTCTCTGCC
H_CALR_F	AGTTCCGGCAAGTTCTACGG
H_CALR_R	ACAGAGCATAAAAGCGTGCAT
H_CAP1_F	GGGGAGACGTGCAGAAACAT
H_CAP1_R	GGGTGCCAACAAATCGGAAAG
H_METTL7A_F	CAGAGTGCTGAGACCGGGA
H_METTL7A_R	CTGGTCAGGTTGCACCCATC
H_ENO1_F	GCCGTGAACGAGAAGTCCTG
H_ENO1_R	ACGCCTGAAGAGACTCGGT
H_UBE2O_F	ACTAGAGGACCGTTCTGTGGT
H_UBE2O_R	TGACGGGATAGATGATGCAGTT
H_PFKL_F	GTACCTGGCGCTGGTATCTG
H_PFKL_R	CCTCTCACACATGAAGTTCTCC
H_DTYMK_F	CCGGTTCCCGGAAAGATCAAC
H_DTYMK_R	TCCCAGCGATTTGCAGAAAAA
H_IDE_F	TTTTCAGCCCATTTGCTTATGTG
H_IDE_R	TGCATACTCGTTGAGTGAGTCTT
H_GSR_F	TGATCCCAAGCCCACAATAGAGGTCAGT
H_GSR_R	CCATCGCTGGTTATTCCTAAGCTGGCAC
H_RPIA_F	AGTGCTGGGAATTGGAAGTGG
H_RPIA_R	GGGAATACAGACGAGGTTCAGA
H_ENSA_F	AGGCAAAGCTAAAGGCCAAAT
H_ENSA_R	GCCATGTTGTAGTCTCCTGAGT
H_HSPB1_F	ACGGTCAAGACCAAGGATGG
H_HSPB1_R	AGCGTGTATTTCCGCGTGA
H_PLIN3_F	TTGCATCAGCCAGCGAATACGC
H_PLIN3_R	CACCTTAGACGACACAAGCTCC
H_CXCL8_F	CACTGCGCCAACACAGAAAT
H_CXCL8_R	GCCCTCTTCAAAAACTTCTCCAC

H_CCL21_F	AGCAGGAACCAAGCTTAGGCTG
H_CCL21_R	GGTGTCTTGTCCAGATGCTGCA
H_CCL2_F	CCCAAAGAAGCTGTGATCTTCA
H_CCL2_R	TCTGGGGAAAGCTAGGGGAA
H_CCL3_F	TGCAACCAGTTCTCTGCATC
H_CCL3_R	TGGCTGCTCGTCTCAAAGTA
